# Influence of Process and Material Factors on the Quality of Machine Processing of Laminated Particleboard

**DOI:** 10.3390/ma18143402

**Published:** 2025-07-21

**Authors:** Łukasz Adamik, Radosław Auriga, Piotr Borysiuk

**Affiliations:** 1 Institute of Wood Sciences and Furniture, Warsaw University of Life Sciences-SGGW, ul. Nowoursynowska 159, 02-776 Warsaw, Poland; radoslaw_auriga@sggw.edu.pl (R.A.); piotr_borysiuk@sggw.edu.pl (P.B.); 2 Nowy Styl sp. z o.o., ul. Pużaka 49, 38-400 Krosno, Poland

**Keywords:** particleboard, machining, machining quality, laminate breakout, delamination, tool wear

## Abstract

Next to solid wood, laminated particleboard is the most widely used wood-based material in the furniture industry. Ensuring the high quality of the laminate surface after machining is of critical importance for furniture manufacturers, particularly prior to the edge banding process, as this process significantly influences the final aesthetic and functional quality of panel elements. The objective of this review article is to gather and evaluate the current state of knowledge regarding the influence of machining process parameters and the physical and mechanical properties of laminated particleboard on machining quality. Particular emphasis is placed on the occurrence of laminate damage, commonly referred to as delamination, a prevalent defect in the furniture manufacturing sector. Both categories of influencing factors—process-related and material-related—are analyzed within the context of the three primary technological processes employed in the woodworking industry, namely drilling, cutting, and milling. The analysis revealed that a persistent research gap concerns the relationship between machining quality and material parameters, particularly in the case of milling—a process of critical importance in the furniture industry.

## 1. Introduction

Particleboards are engineered wood materials composed of wood chips bonded with a resin-based adhesive. These chips are formed into a mat and compressed to achieve the target thickness [1]. In subsequent processing stages, a decorative surface layer is often applied through pressing. This typically involves the lamination of various materials, most commonly melamine resin-impregnated paper. Following this lamination process, the particleboard is referred to as melamine-faced chipboard (MFC). Melamine-coated boards are widely used in furniture manufacturing, particularly for cabinets, desks, tables, beds, dressers, and other similar applications.

The value of global particleboard production in 2022 was USD 22.4 billion and is expected to reach USD 30.95 billion in 2031 [2]. In 2023, the market value was estimated at USD 23.0 billion, with projections indicating that it will reach USD 31.3 billion by 2032 [3]. A notable proportion of particleboard undergoes further processing through coating with laminates. However, current data on the exact share of this finishing process remains absent from both the scientific literature and online sources. In the past, this share has been as high as 70% [4] of annual production. Other sources report that, in 2022, the value of global production of laminated panels, manufactured with melamine resin, was USD 18.6 billion. It is expected to reach USD 31.26 billion in 2030 [5]. These values, however, are distorted by the presence of MDF board in these data, which is also covered with melamine formaldehyde resin-impregnated paper. This material is less common in the industry, but, on the other hand, it does have an impact on the value of global production, and it is difficult to determine, based on that data, what the exact share of laminated particleboard is.

**Figure 1:**
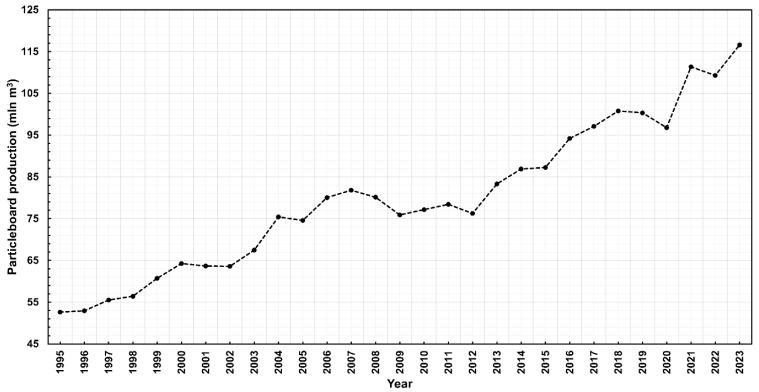
Global particleboard production 1995–2023 (million m^3^).

**Figure 2:**
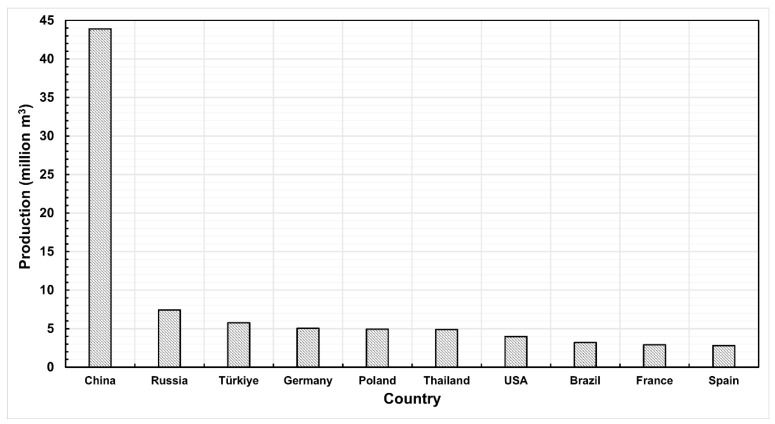
Global particleboard production by country in 2023.

**Figure 3:**
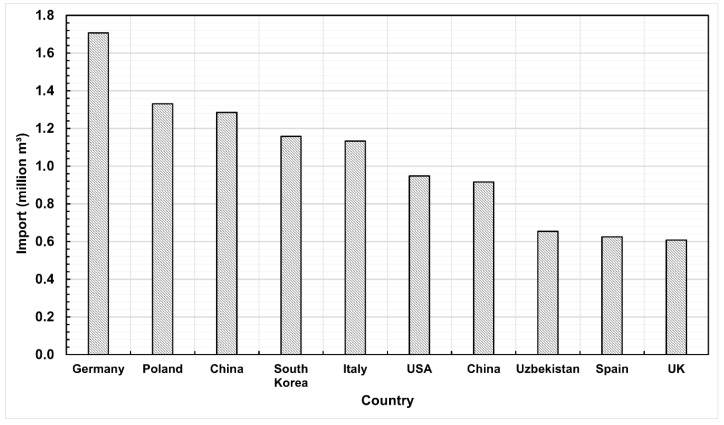
Largest particleboard importers by country in 2023.

In 2023, over 116 million square meters of particleboard were produced worldwide, and global production continues to show an upward trend (Figure 1). Poland, as the third-largest furniture exporter in the world [6], is also an important consumer of the primary furniture material, which is particleboard. Its supply comes from two sources. The first is domestic production, where in 2023, the production of particleboard amounted to 4,936,312 m^3^, placing Poland fifth among the largest producers of particleboard globally (Figure 2). Secondly, the needs of Polish furniture manufacturers are supplemented through the import of this material into the country. In 2023, Poland was the second-largest importer of particleboard in the world, with an import volume of 1,331,515 m^3^ (Figure 3) [7].

**Figure 4:**
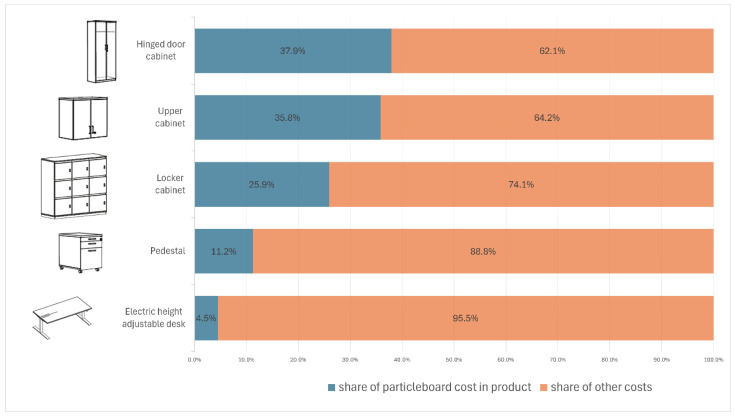
Examples of the proportion of particleboard in the cost of representative products.

Particleboard is a fundamental construction material for many products in the woodworking industry, including furniture (residential, kitchen, office, and public use), window and door production, flooring, and many others. Due to its good machinability and advanced processing technologies, the material allows for rapid shaping and sizing of the panel components, as well as edge banding and perforation. In furniture products, particleboard represents a significant volumetric and mass share, but, more notably, it plays a critical role in production cost. For instance, in the office furniture sector, the proportion of particleboard in the cost of a product can reach as much as 40% in certain items (Figure 4); the data come from internal sources of one of the largest office furniture producers. Such a high share of this material in terms of cost makes machining and the quality of the cutting process key factors due to their significant impact not only on the price of furniture products but also on their perception and evaluation by the customer.

Another aspect that determines the important role of the quality of wood-based panel machining is the ongoing trend toward increased factory automation and the broader implementation of Industry 4.0 solutions in manufacturing facilities [8]. Modern furniture factories, also known as smart factories [9], not only execute technological processes without human intervention but also manage transportation and inter-operational tasks. These factories are capable of automatic setup and retooling for workpieces delivered randomly within the production process. Furthermore, through real-time production analysis (based on established priorities), the software autonomously makes managerial decisions regarding production pace, generated technological waste, process costs, and even the quality level of the manufactured components. In such production systems, a stable technological process requires a high degree of predictability of the factors influencing quality. The extent of understanding their impact and the level of control over these factors determines the alignment of products with customer requirements, which directly translates into the business success of furniture companies.

The high level of global production of laminated particleboard, its significance as a construction material, and its considerable influence on the technological processes of large wood industry factories underscore the importance of maintaining high quality of this material in terms of machining. This paper presents the current state of knowledge regarding machining processes and the factors influencing them. These factors have been categorized into those originating from the process and those arising from the material. In the literature, these phenomena are characterized as a material–process–quality model [10].

## 2. Materials and Methods

In the context of this study, it was necessary to identify an appropriate and effective method for conducting a literature review. Due to the large volume of articles on the topic of machining quality and the differences in content across various electronic databases of scientific publications, a systematic literature review was chosen for this purpose. Scopus and the Web of Science were analyzed as the two most reliable and relevant article databases. Additionally, due to the high number of articles retrieved and to obtain a broader perspective, the Google Scholar database was also consulted. The gray literature was excluded from the analysis to ensure methodological rigor.

**Figure 5:**
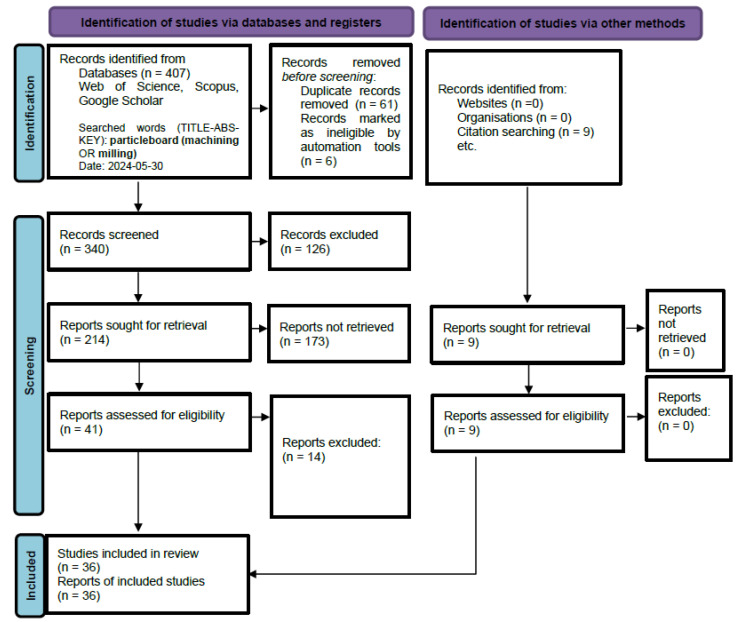
PRISMA diagram—systematic literature review.

For the SCOPUS database, the query phrase was as follows: TITLE-ABS-KEY (particleboard AND machining OR particleboard AND milling). For the Web of Science database, the query was as follows: particleboard (machining OR milling). For Google Scholar, it was “particleboard machining” OR “particleboard milling”. A total of 407 articles were found across the 3 databases, which, after removing duplicates, resulted in 340 publications. After reviewing and screening, 41 papers were selected for the review article. Additionally, during a thorough examination of these articles, 9 more publications were found based on citations. The literature review is graphically presented in the PRISMA diagram (Figure 5). This systematic review was registered on the INPLASY platform (International Platform of Registered Systematic Review and Meta-analysis Protocols) under the registration number INPLASY202570039. The protocol is publicly available at https://doi.org/10.37766/inplasy2025.7.0039.

It should be noted that many articles have investigated the impact of technological and material factors, primarily on the physical properties of particleboards, such as mechanical strengths (mainly bending and tensile strength), and often also on cutting forces. However, when it comes to the direct impact on cutting quality and machinability of laminated particleboards, the number of articles is limited. In particular, there is a low number of articles addressing the influence of material factors. The small number of such studies suggests a gap in previous research and opens an interesting area for further scientific exploration.

## 3. Laminated Particleboard Machining Quality

To assess the quality of machining laminated particleboards, it is essential to first define the physical parameters that represent this quality. A systematic literature review identified three such parameters. First, surface roughness after machining can be considered [11]. This parameter indeed describes a qualitative aspect, as the relationship is such that the higher the roughness, the lower the quality. However, it does not have a direct correlation with the aesthetic appearance of the panel element. It appears to be relevant when applied to raw materials, such as solid wood, plywood, or raw particleboard. For elements coated with paint or laminate, it would be unrepresentative and inadequate. In the evaluation of a furniture component or an entire product (furniture) by the user, the appearance of the edges, hole edges, or milling is of greater importance.

Therefore, the area of delamination or laminate damage is more commonly used in the literature to describe the quality of machining coated panels. In other words, it refers to the surface area of the panel element where laminate (on the decorative surface) should be present but has been improperly removed by the cutting process. As early as 1984, this method was characterized as highly effective and appropriate for machining, particularly for the edge milling of components [12].

A specific case is the drilling process. Within this technology, some studies define quality by the height of the laminate lift above its proper surface [13]. This method is of significance not only for laminated panels but also for horizontal drilling in edges covered with edge materials, such as ABS or PP. This has substantial practical importance, as the drilled hole, creating a collar of unwanted extra material, can result in improper alignment of the furniture component with the accompanying part. An example of this can be the connection of the upper cabinet top shelf with the side wall using a dowel joint, where the extra material causes the presence of a gap between the elements.

**Figure 6:**
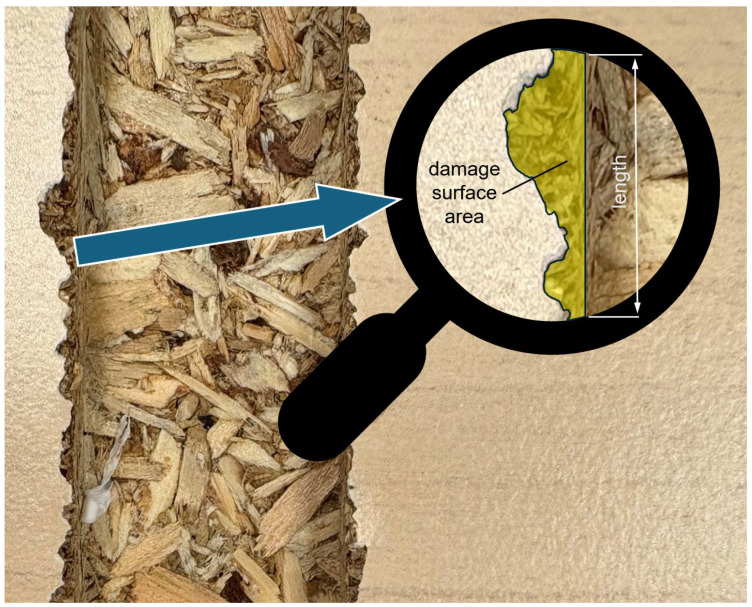
Damage surface area referenced to the length quality indicator [14].

Due to the effectiveness, significance, and widespread use of methods describing the area of delamination, this issue requires further explanation. First, a simple and effective method is the delamination area referenced to the length of the path being machined by the tool. The nominal edge line is determined by the points where the laminate surface meets the edge of the machined material. Any deviation from this line indicates a loss of laminate or even the particleboard itself. The total area of these deviations, referenced to the measured length, defines the discussed indicator, quality indicator = damage area/length [14], most often represented in units of mm^2^/m (Figure 6).

**Figure 7:**
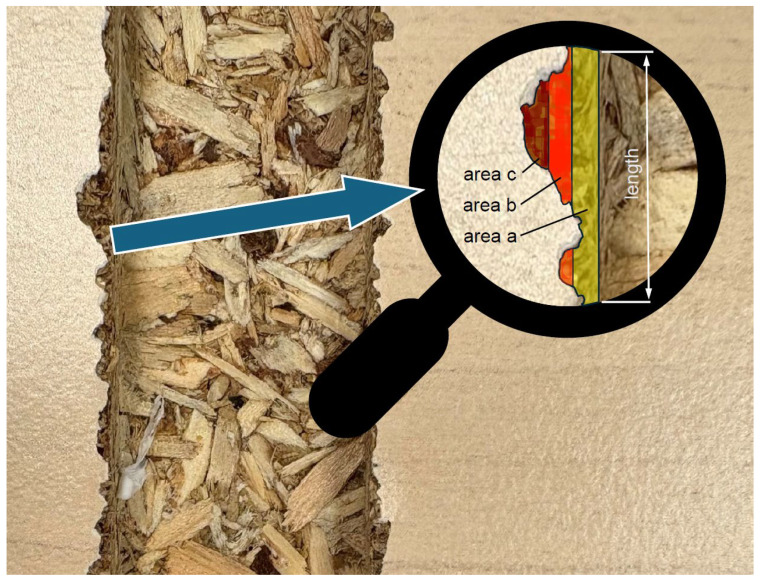
Weighted damage surface area referenced to the length quality indicator [12].

A more advanced method for assessing the quality of machining involves taking into account the depth of delamination. German researchers [12] have observed that deeper damage penetration has a greater impact on negative visual perception, particularly at furniture panel edges. Therefore, they introduced weighting factors into the method for calculating the quality indicator. The greater the penetration of the defect, the higher its influence on the indicator (Figure 7); a representative formula for this indicator is as follows: quality indicator = (area a·1 + area b·2 + area c·5)/length [mm^2^/m]. In later studies on the cutting of particleboards, this method was further developed and refined [15]. First, the reference line was changed from the mean profile line to the maximum profile line, as the maximum line represents the position where the furniture edge banding material is attached, a critical area for panel components. Second, the method of weighting areas was modified from a discrete to a continuous approach by employing a quadratic function for weights. In this function, the significance of delamination occurring below the limit value is reduced, while areas exceeding the limit value result in a rapid increase in the quality indicator.

**Figure 8:**
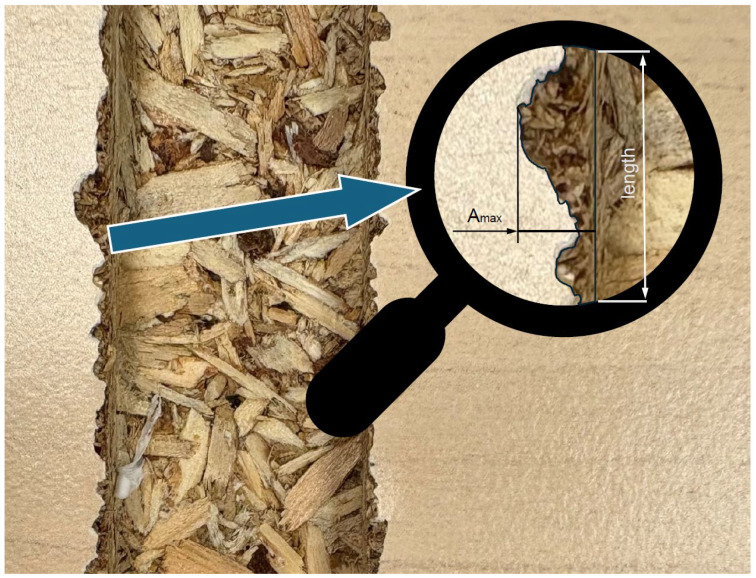
Max damage depth quality indicator [16].

Another simplified quality indicator is the determination of the maximum damage along the inspected section [16], expressed in units of length, such as millimeters (Figure 8), with the following formula: quality indicator = A_max_. The advantage of this method is its ease of application and measurement, but its major drawback is its sensitivity to anomalies and large individual damages. Therefore, the method is effective for high-quality boards, tools with low wear levels, or for quick tests in industrial facilities. In the case of drilling, a variation of this indicator involves measuring the hole diameter up to the maximum delamination point. This value is then taken away from the nominal diameter and divided by two [17].

## 4. Process Factors

The key question that this study aims to address is which factors influence the previously presented quality indicators, how they do so, what the sources of defects and poor machining quality are, and which parameters contribute to its improvement. First, factors originating from the machining process will be discussed. Due to the varying nature of different types of machining, these factors have been further categorized based on the processes in which their impact on the final quality has been investigated. The literature provides information on three primary machining processes for particleboards, namely drilling, cutting, and milling. The process factors, along with a brief description of their impact, are summarized in Table 1.

### 4.1. Drilling

Drilling, as a machining technology, and more specifically the resulting holes, might initially seem less significant from a quality perspective, particularly regarding delamination defects (as holes are often covered by other panel components or by furniture fittings, such as rosettes for locks, plastic screw cap covers etc.). However, this view is incorrect, as holes are frequently exposed to the user—for instance, shelf position holes, holes for furniture handles, and holes for hinges. A statistical review of the literature has identified the following investigated factors.

For drilling technology, a relationship has been demonstrated between cutting parameter values, tool wear, and the surface quality of laminated particleboards after machining. It has been proven that cutting speed and tool wear have the most significant impact on this quality. Moreover, as the feed rate and cutting speed increase, tool wear also increases. Tool wear contributes to an increased delamination area on the particleboard [18]. In this study, the analysis focused on through-hole drills, with the entry and exit points of the tool analyzed separately. This distinction is of practical importance due to differing quality requirements for the right and left sides of furniture components.

In the drilling process, the parameters considered to have a decisive influence on machining quality are the axial force and cutting torque. Given this, the influence of other cutting parameters on these two variables is demonstrated. A known relationship exists between the feed per revolution and the axial force, where an increase in feed results in an increase in axial force. Additionally, as tool wear increases, both the cutting torque and axial force also increase [19].

Due to these correlations, an intriguing proposal involves adjusting cutting parameters based on the characteristics of the material being machined. Researchers [20] suggest increasing feed rates or cutting speeds for materials with higher machinability and resistance to machining during drilling. This approach is groundbreaking, given current technological capabilities and the Industry 4.0 solutions employed in the industry. The proposed solutions enable real-time monitoring of the machined material and dynamic regulation of cutting parameters in response to its characteristics. A simplified proposal for assessing material machinability involves the test drilling of a given material under constant loads (3, 2.75, and 2.5 kg) and measuring the time required to drill a hole to a specified depth, which serves as the evaluated indicator [30].

Advanced research is also being conducted in the area of drill bit surface modification, aimed at increasing tool life. For this purpose, ion implantation (using nitrogen ions) has been applied, and an increase in tool life has been demonstrated through tests conducted during the drilling of laminated particleboards [21]. These studies have initiated significant theoretical discussions regarding the processes occurring during machining and the dynamic phenomena of surfactant film formation and destruction, which could potentially lead to infinite tool durability, particularly in the machining of cellulose-based materials, such as wood and particleboards [31].

### 4.2. Cutting

Cutting, as a process of dividing large-format commercial boards into smaller furniture components with required design dimensions and preparing them for the edge banding process, is also a significant technology in terms of quality. In this case, it primarily concerns the edges of the boards, or the grooves cut into them in other further processes.

In the case of cutting, detailed studies were conducted [15], confirming a decrease in quality with increasing tool wear. Additionally, the influence of various factors was comprehensively analyzed using three quality indicators (mentioned in Section 3)—delamination area referenced to the length of the path indicator, the damage depth weighted delamination quality indicator, and the maximum depth of laminate damage indicator. This approach allowed for a more thorough understanding of the machining quality phenomenon during cutting. A practically significant industrial relationship was demonstrated (with the weighted indicator)—as cutting speed increases, the quality indicator may decrease (indicating an improvement in quality). Moreover, the higher the saw wear, the more effectively quality can be improved by increasing the cutting speed, particularly in terms of reducing wide laminate damages.

A specific characteristic of the cutting process for laminated particleboard is the significant importance of material support [15]. In industrial practices, this support is typically provided by the machine’s worktable. Unfortunately, achieving complete and effective support solely with the worktable is very difficult or even technically impossible, particularly at the tool’s exit point from the material. For this reason, this technology employs a scoring saw, which cuts into the laminated board from the opposite side being processed and operates with reverse rotation to the main saw. Its function is to make a shallow cut, usually about 2 mm deep, with a kerf width slightly larger than that of the main saw. This ensures that the material susceptible to damage is not present in the area where the main saw exits [32].

Among other relationships in the sawing process, it has been demonstrated that an increase in the feed per tooth results in a rise in the quality indicator (indicating a decline in quality). In other words, the more material is cut by a single tooth, the poorer the quality. The same conclusions were drawn regarding the relationship between the quality indicator and feed speed. Additionally, it was found that the greater the amount of energy transferred to the cutting process, the better the quality of the results achieved [22].

### 4.3. Milling

Among the factors affecting the machining quality of laminated boards that originate from the process, milling appears to be the best-documented. This may be due to the increasing significance of this technology in furniture production. Currently, in the edge banding process, the attachment of the furniture edge banding material to the panel is preceded by pre-milling. This operation serves two purposes: first, to prepare the board’s edge for adhesion with the glue, and second, to align the geometry of the component relative to the edge-banding machine (referencing). Through the use of the aforementioned pre-milling, milling quality becomes critical in industrial production, as it determines the final aesthetic value of the furniture component (including the presence or absence of laminate damage around all the banded edges).

Similarly to cutting and drilling processes, quality indicators increase (the area of laminate damage grows, thereby reducing quality) with tool wear [10] as well as with decreasing cutting speed. These findings have been corroborated by other studies; furthermore, the depth of the milled particleboard layer also plays a significant role in quality [23]. The investigations did not demonstrate any correlation between the feed per tooth and changes in quality indicators [33].

The quality of machining is also dependent on the material from which the tool’s cutting edge is made. Studies have demonstrated that the cutting edge of a high-speed steel milling tool, when compared to a carbide milling tool, exhibits two key differences. Firstly, at the same level of wear, the high-speed steel tool shows a higher delamination factor (indicating lower quality). Secondly, it degrades more rapidly over time, leading to faster wear and, consequently, a quicker decline in cutting quality [24]. Further research has revealed that the specific type of carbide used (its hardness and nanoscale elastic modulus) significantly affects the tool’s durability and, as a result, the overall quality of machining [25]. It is not only the material but also the tool’s geometry that influences machining quality. It has been proven that for optimal milling quality, a tool with the smallest possible cutting angle should be used. However, caution is necessary, as excessively small cutting angles may result in a shortened tool life due to accelerated wear. Optimization in this context requires a balance between these two parameters [26].

Experiments were also designed to study energy consumption during the milling of particleboards with varying physical properties. The objective was to develop a classification of board types and optimize feed rate values (maximization), while simultaneously minimizing tool wear, ultimately leading to a reduction in process costs. The experiment did not reveal a clear relationship between energy consumption and the material’s properties but did demonstrate changes in the material’s physical properties along the milling path within the same tested material [34].

The impact of technological parameters on milling is so well-documented that they have been incorporated into the diagnostics of this process. Researchers [27], by identifying relationships between the quality coefficient and process parameters (cutting forces, tool wear, and the work required for milling), confirmed that by using neural networks (with elements of fuzzy logic algorithms), it is possible to predict the machining outcome of particleboards.

The scientific literature also features numerous publications on the modification of cutting tool surfaces in milling cutters, aimed at extending tool life and indirectly improving the quality of the particleboard milling process. One proposed approach involves applying a coating using the submerged arc-welding method (SAW), which has been shown to increase tool wear resistance during the milling of wood-based materials [28]. Another surface modification technique employed by researchers [35] is the use of extreme ultraviolet lithography (EUV), utilizing intense ultraviolet radiation pulses with a laser as the irradiation source. However, in this case, the results did not confirm an extension of the tool’s life. A further method is ion implantation, previously described in the context of drilling processes. Research findings demonstrated a significant increase in tool life for WC–Co carbide cutting edges used in milling tools for particleboards [29].

## 5. Material Factors

The second equally significant source of factors affecting the quality of machining is, naturally, the material being processed. From a practical (industrial) perspective, this source is more difficult to control, as the processed material is typically a purchased component for furniture manufacturers, and only a few producers manufacture particleboards for their own internal use. However, understanding these factors allows for effective communication with suppliers and the establishment of quality specifications for the materials delivered.

In the scientific domain, advanced models have been developed to simulate the physical phenomena occurring during the cutting of particleboards, demonstrating the interaction between the cutting tool and the processed material [36], later expanded to include qualitative aspects of particleboard machining [37]. These studies indicated that when the bonding strength of the chips with the adhesive is higher than the chip’s internal strength, the quality of the machined edge is high (satisfactory). Conversely, when the adhesive bonding is weak, chip detachment (laminate damage) occurs. The simulations also defined a relationship where an increase in cutting forces is associated with greater cutting depth, higher adhesive content, and higher chip strength. Additionally, it was determined that as the tool rake angle increases, these forces decrease. It was also demonstrated that the bonding strength within the particleboard can be increased by optimizing the chip geometry or by enhancing the adhesive’s bonding force, which directly improves the cutting quality of the board. It should be noted that these studies were preceded by simulations with greater practical relevance, which confirmed that the lack of proper laminate support, voids within the board, or the presence of bark are critical factors during the cutting process [15].

As in the previous chapter, other factors have been presented separately for the three most commonly used technological processes. A statistical review of the literature revealed the following state of knowledge. The material factors, along with a brief description of their impact, are summarized in Table 2.

### 5.1. Drilling

Regarding the impact of material factors on the drilling process, the available literature primarily focuses on the raw materials used in the boards (e.g., the wood species of the chips and the addition of synthetic materials). Furthermore, it appears that these studies are predominantly comparative analyses involving industrially used particleboards. This focus is driven by the current trend and the need to design new wood-based materials incorporating alternative raw materials to increase the utilization of by-products, thereby contributing to the development of environmentally friendly solutions in these materials.

An example of such a raw material is the fruit tree branches obtained from orchard pruning. It has been found that particleboards made from apple tree branches exhibit higher axial force and cutting torque compared to reference boards (pine chips) and boards produced from plum tree branches. In contrast, boards made from plum tree branches demonstrate properties similar to those of the reference board [38]. Another material used is the addition of grapevine branches, where it has been proven that their presence reduces the axial force during drilling [39].

Another possible addition to particleboards are synthetic materials, such as PP (polypropylene), PE (polyethylene), and PS (polystyrene). It has been confirmed that both the type of material used as an additive and its content within the wood composite influence the drilling quality on the tool entry surface. However, for the opposite side (the drill exit surface) of the board, only the type of synthetic material plays a significant role. The general rule is as follows—the higher the content of the synthetic material, the better the drilling quality on the tool entry side [17].

### 5.2. Cutting

Extensive research on the cutting quality of particleboards has been conducted, utilizing computer simulations based on the finite element method (FEM) [40]. After constructing a theoretical simulation model, the stress distribution was analyzed at the point of tool penetration during the cutting of the particleboard’s outer layer (melamine paper). Two models were simulated [41]—the first had the melamine coating properly supported by wood chips, while the second had it was supported by bark or had an empty space beneath it. It was demonstrated that a lack of proper support causes high stresses in the decorative layer (laminate), leading to laminate defects. These studies also confirmed that the tool’s edge rounding radius adversely affects cutting quality and generates higher stresses compared to a tool with a small edge rounding radius (a sharp tool). The research emphasizes the critical role of edge cutting as a key area influencing the quality of final furniture components.

Another material factor studied in the cutting process is the raw material used in board production. Research has shown that the addition (50%) of alternative raw materials, such as straw, acacia wood chips, or willow wood chips, reduces cutting forces during mechanical processing compared to the reference board produced from standard raw materials. At the same time, the boards maintain their favorable physical and mechanical properties [42].

### 5.3. Milling

The literature highlights several factors related to the processed material that can influence the milling process and its quality. The first significant factor identified is the mineral content (sand) in particleboard [43]. This parameter has an indirect impact on quality, as it accelerates tool wear during processing, which subsequently leads to a decline in milling quality. Another factor associated with the material is the content of thermoplastics added to the particleboard. Additions of 30% and 50% polystyrene and polypropylene positively affected the tool’s life, resulting in improved machining quality for these boards [44]. Another conclusion from these studies was the reduction in cutting forces and cutting torque compared to commonly used boards, indicating improved machinability.

From the perspective of material factors, researchers emphasize the characteristics of surface layers, specifically laminate layers. It has been demonstrated that there is a correlation between the quality achieved during milling (quality indicators) and the force measured during the laminate pull-off test. The higher the force required to pull off the laminate during testing, the better the quality indicator (higher quality) achieved later during the machining of laminated boards [45]. Furthermore, coating the surfaces also contributes to quality improvement [16]. Better quality indicator results are achieved by laminating or coating furniture boards with varnish.

## 6. Conclusions

After a detailed review of the literature and confirmation of the significance and scale of furniture production from laminated particleboards, it is essential to acknowledge the high necessity of understanding the factors influencing the machining quality of these boards. While process-related factors (primarily those arising from the operation and technological parameters of the tool) have been extensively studied for all major processes—drilling, cutting, and milling—material-related factors appear to be a subject requiring further scientific investigation.

In particular, the aspect that represents a research gap concerns material-related factors influencing the milling process. A systematic literature review identified only four publications that thoroughly analyzed these phenomena and directly addressed the milling process, with two of them published before 2010, suggesting a lack of recent studies.

From a practical (industrial) perspective, milling is currently a key process in furniture production. It determines the final quality of panel components and, consequently, the finished piece of furniture. This is a result of the construction and technology used in furniture manufacturing. More specifically, milling is not only used for machining pockets and holes for furniture fittings but also plays a crucial role as a preparatory process between the cutting of laminated particleboard and the edge banding operation. In most industrial edge banding machines, the first processing zone contains formatting heads, often preceded by shredders, which remove a small amount of the particleboard material (typically 1–3 mm per side). This step prepares the edge for strong adhesion with the glue, removes contaminants, and ensures proper alignment of the panel’s edge with the machine’s processing path. This alignment is essential for the effective operation of subsequent processing units, including edge banding material trimmers, scrapers, and polishers. Ultimately, this process impacts the quality of the furniture component, particularly in terms of the quality and appearance of the applied edge banding (ABS, PP, PMMA) or natural wood edging.

Moreover, practical experience from industrial plants indicates that, under constant technological parameters, different types of boards exhibit varying tendencies for machining defects, primarily delamination. This aspect serves as both a reason and a motivation for further research to address the following questions: which material factor has the greatest influence on this tendency, how can these factors be assessed in an industrial setting, and how can manufacturers safeguard against lower-quality materials? Additionally, the ability to identify lower-quality material enables its exclusion from the production process. Consequently, this leads to cost savings through a reduction in production waste and the minimization of material requiring disposal, which would otherwise have a negative impact on the environment. This, in turn, allows companies to enhance their competitiveness, demonstrate a commitment to sustainability, and achieve better outcomes in life cycle assessment (LCA) evaluations of furniture products.

## Figures and Tables

**Table 1:** Summary of process factors influencing the cutting quality of laminated particleboard.

Author (Year)	Source of Influence	Process	Factor	Main Findings	Reference
Szwajka et al., 2017	Process	Drilling	Tool wear, cutting speed	When feed rate and cutting speed increase, tool wear also increases. Tool wear contributes to an increased delamination area on the particleboard	[18]
Zielińska-Szwajka et al., 2014	Process	Drilling	Axial force, cutting torque	Increase in feed results in an increase in axial force As tool wear increases, both the cutting torque and axial force also increase	[19]
Szwajka et al., 2024	Process	Drilling	Feed rate, cutting speed	Adjusting cutting parameters based on the characteristics of the material being machined	[20]
Wilkowski et al., 2022	Process	Drilling	Drill bit surface modification	Ion implantation (using nitrogen ions) has been applied, and an increase in tool life has been demonstrated	[21]
Pałubicki 2006	Process	Cutting	Tool wear, cutting speed, material support	As cutting speed increases, the quality indicator may decrease (indicating an improvement in quality)	[15]
Garrido et al., 2024	Process	Cutting	Feed per tooth, feed speed, amount of energy transferred to the process	Increase in the feed per tooth and in the feed speed results in a rise in the quality indicator (indicating a decline in quality) The greater the amount of energy transferred to the cutting process, the better the quality of the results achieved	[22]
Kowaluk et al., 2007	Process	Milling	Tool wear, cutting speed	Quality indicators increase (the area of laminate damage grows, thereby reducing quality) with tool wear as well as with decreasing cutting speed	[10]
Li Hao et al., 2022	Process	Milling	Depth of the milled particleboard layer	As the milling depth increases, the processing quality decreases	[23]
Szwajka et al., 2016	Process	Milling	Tool’s cutting edge material	The high-speed steel tool shows a higher delamination factor (indicating lower quality) compared to the carbide tool The high-speed steel tool degrades more rapidly over time, leading to faster wear and, consequently, a quicker decline in cutting quality	[24]
Wilkowski et al., 2019	Process	Milling	Tool’s cutting edge material	Type of carbide used (its hardness and nanoscale elastic modulus) significantly affects the tool’s durability	[25]
Kowaluk et al., 2009	Process	Milling	Tool’s geometry	For optimal milling quality, a tool with the smallest possible cutting angle should be used. However, excessively small cutting angles may result in a shortened tool life due to accelerated wear	[26]
Lacki et al., 2009	Process	Milling	Cutting forces, tool wear, work required for milling	By identifying relationships between the quality coefficient and process parameters (cutting forces, tool wear, and the work required for milling), it was confirmed that by using neural networks, it is possible to predict the machining outcome of particleboards	[27]
Bendikiene et al., 2016	Process	Milling	Modification of cutting tool surfaces	Applying a coating using the submerged arc-welding method (SAW), has been shown to increase tool wear resistance during the milling of wood-based materials	[28]
Wilkowski et al., 2022	Process	Milling	Modification of cutting tool surfaces	Using ion implantation, an increase in tool life for WC–Co carbide cutting edges used in milling tools for particleboards was demonstrated	[29]

**Table 2:** Summary of material factors influencing the cutting quality of laminated particleboard.

Author (Year)	Source of Influence	Process	Factor	Main Findings	Reference
Wong 2007	Material	All processes	Particleboard’s bonding strength	Studies indicated that when the bonding strength of the chips with the adhesive is higher than the chip’s internal strength, the quality of the machined edge is high	[37]
Kowaluk et al., 2020	Material	Drilling	Raw material for particleboard production	It has been found that particleboards made from apple tree branches exhibit higher axial force and cutting torque compared to standard boards (pine chips) and boards produced from plum tree branches. Boards made from plum tree branches demonstrate properties similar to those of the standard board	[38]
Auriga et al., 2022	Material	Drilling	Raw material for particleboard production	The addition of grapevine branches has a positive impact; it has been proven that their presence reduces the axial force during drilling	[39]
Górski et al., 2022	Material	Drilling	Raw material for particleboard production	It has been found that the addition of synthetic materials to particleboards, such as PP (polypropylene), PE (polyethylene), and PS (polystyrene) influence the drilling quality on the tool entry surface	[17]
Pałubicki 2006 Pałubicki et al., 2004 Pałubicki et al., 2008	Material	Cutting	Laminate support, voids within the board, presence of bark	Lack of proper laminate support, voids within the board, or the presence of bark are critical factors during the cutting process	[15] [40] [41]
Kowaluk et al., 2007	Material	Cutting	Raw material for particleboard production	The addition (50%) of alternative raw materials, such as straw, acacia wood chips, or willow wood chips, reduces cutting forces during mechanical processing compared to the reference board produced from standard raw materials. At the same time, the boards maintain their favorable physical and mechanical properties	[42]
Porankiewicz et al., 1993	Material	Milling	Mineral content (sand)	Mineral content (sand) has an indirect impact on quality, as it accelerates tool wear during processing, which subsequently leads to a decline in milling quality	[43]
Zbieć et al., 2012	Material	Milling	Raw material for particleboard production	Additions of 30% and 50% polystyrene and polypropylene positively affected the tool’s life, resulting in improved machining quality of such boards	[44]
Porankiewicz et al., 2001	Material	Milling	Laminate adhesion to particleboard	The higher the force required to pull off the laminate during testing, the better the quality indicator (higher quality) achieved later during the machining of laminated boards	[45]
Szymanowski et al., 2015	Material	Milling	Type of coating on particleboard	Coating the surfaces contributes to quality improvement; better quality indicator results are achieved by laminating or coating furniture boards with varnish	[16]

## Data Availability

No new data were created or analyzed in this study. Data sharing is not applicable to this article.
